# Early life stress caused glucose homeostasis impairment and anxiety-like behavior along with oxidative stress in adolescent male rat offspring: Protective effect of saffron extract

**DOI:** 10.1371/journal.pone.0359395

**Published:** 2026-10-07

**Authors:** Siavash Shirzadeh Barough, Behnam Saeidi, Fateme Binayi, Mina Salimi, Fatemeh Siavoshi, Roxana Karbaschi, Mehdi Hedayati, Homeira Zardooz

**Affiliations:** 1 Neurophysiology Research Center, Institute of Neuroscience and Cognition, Shahid Beheshti University of Medical Sciences, Tehran, Iran; 2 Foot and Mouth Disease Reference Laboratory, Razi Vaccine and Serum Research Institute, Agricultural Research Education and Extension Organization (AREEO), Karaj, Iran; 3 Gastroenterology and Liver Diseases Research Center, Research Institute for Gastroenterology and Liver Diseases, Shahid Beheshti University of Medical Sciences, Tehran, Iran; 4 Traditional Medicine and Hydrotherapy Research Center, Ardabil University of Medical Sciences, Ardabil, Iran; 5 Department of Physiology, School of Medicine, Ardabil University of Medical Sciences, Ardabil, Iran; 6 Brain Mapping Research Center, Shahid Beheshti University of Medical Sciences, Tehran, Iran; 7 Faculty of Nursing and Midwifery, Shahid Beheshti University of Medical Sciences, Tehran, Iran; 8 Cellular and Molecular Endocrine Research Center, Research Institute for Endocrine Molecular Biology, Research Institute for Endocrine Sciences, Shahid Beheshti University of Medical Sciences, Tehran, Iran; 9 Department of Physiology, School of Medicine, Shahid Beheshti University of Medical Sciences, Tehran, Iran; Semnan University of Medical Sciences and Health Services, IRAN, ISLAMIC REPUBLIC OF

## Abstract

Early life stress (ELS) significantly contributes to metabolic and psychological disorders by triggering oxidative stress and disrupting the hypothalamic–pituitary–adrenal (HPA) axis. This research examined ELS effects on redox status, along with anxiety-like behavior, in adolescent male Wistar rats, also assessing saffron (Crocus sativus L.) extract’s potential benefits. Rats were exposed to foot shock stress from postnatal days 15–19, with treatment groups receiving saline or saffron extract (10 mg/kg) via intraperitoneal injection. Control groups were maintained without stress exposure. Elevated Plus Maze (EPM) and Open Field Test (OFT) were used to evaluate anxiety-like behaviors. Corticosterone, glucose, and insulin levels, oxidative stress markers [malondialdehyde (MDA), glutathione (GSH), and catalase activity] in the plasma, pancreas, and amygdala were determined, and the HOMA-β index was estimated. Saffron extract markedly restored ELS effects by reducing fasting plasma glucose and corticosterone levels, while increasing insulin levels and the HOMA-β index in adolescent offspring. Additionally, saffron extract inhibited oxidative stress, evidenced by reduced MDA, normalized catalase activity, and augmented GSH levels in the plasma, pancreas, and amygdala. Behaviorally, saffron extract-treated rats exhibited diminished anxiety-like behaviors, characterized by increased time spent in the open arms of the EPM and the center zone in the OFT. This study suggests that ELS induces disturbances in glucose metabolism and anxiety-like behavior, accompanied by oxidative stress and increased HPA axis activity. The protective role of saffron extract underscores its capability to serve as a medicinal compound for preventing and treating stress-induced metabolic and anxiety disorders.

## Introduction

An unhealthy lifestyle and exposure to harmful environmental factors are linked to numerous diseases, as they promote oxidative stress, which occurs due to a disproportion between free radicals and antioxidants within the body [1]. According to our previous studies, chronic feeding of a high-fat diet (as an unhealthy lifestyle) after weaning could cause endoplasmic reticulum stress (ER stress) and oxidative stress in organs such as the pancreas [2] and liver [3] in adult Wistar rats. Studies conducted on both humans and animals have shown that early life stress (ELS) significantly increases the risk of future oxidative stress–related metabolic and psychological disorders in adulthood, including metabolic syndrome, diabetes, anxiety, depression, and cognitive impairments [4,5]. ELS paradigms are commonly classified into maternal separation and early aversive or unpredictable stress models. Maternal separation primarily induces early-life deprivation via disruption of maternal care, whereas neonatal foot-shock and other unpredictable stress paradigms model discrete, uncontrollable adverse experiences during critical developmental periods [6,7]. These models consistently produce long-term alterations in emotional behavior, including increased anxiety-like behavior and dysregulation of the hypothalamic–pituitary–adrenal (HPA) axis [7–9], accompanied by persistent changes in limbic regions such as the amygdala and hippocampus [8,9]. A key advantage of early life aversive stress paradigms is their reproducibility and ability to induce stress independent of maternal care variability, a limitation of separation-based models [10,11], enabling more precise assessment of early life stress–induced neurobiological programming. Some regions of the brain, like the amygdala, are vulnerable and sensitive to ELS and can increase the risk for later psychopathology, such as anxiety-related behavior [12]. Moreover, there is a relationship between ELS, obesity, and metabolic disorders. In this regard, some studies have shown that ELS is linked to increased activity of HPA axis and increased levels of glucocorticoids (mainly corticosterone), which can lead to metabolic abnormalities such as diabetes and heart-related diseases in later stages of life [13,14]. Furthermore, direct insulin infusion into the CeA acutely suppresses feeding via a PI3K-dependent decrease in neuropeptide Y (NPY) and an increase in oxytocin mRNA, which has potent anxiolytic effects [15]. The HPA axis hyperactivity could lead to cellular oxidative stress and oxidative stress-related abnormalities like metabolic disorders, autoimmune diseases, malignancies, and psychological disorders [16]. Chronic HFD (as a metabolic stressor) induced oxidative stress, increased plasma glucose concentration, and reduced insulin and HOMA-β index [2]. Therefore, since oxidative stress plays a crucial role in the development of numerous diseases, the use of antioxidant supplements, especially those derived from plants with antioxidant properties, could contribute positively to both the prevention and treatment of these diseases. Saffron (*Crocus sativus* L.) is known for its high levels of antioxidants and anti-inflammatory benefits and has been utilized in traditional medicine for thousands of years as a natural remedy to combat oxidative stress and promote overall health [17]. Oral consumption of saffron extract has been shown to reduce oxidative stress parameters [18]. Saffron improves depression and impairments of spatial learning and memory caused by oxidative stress in animals [19]. Based on the studies mentioned above and the established relationship between metabolic and behavioral disorders, we explored the impact of saffron extract on metabolic and behavioral problems, along with oxidative stress, resulting from ELS. To investigate this, we assessed the effect of early-life foot shock stress on glucose metabolism, anxiety-like behaviors, and oxidative stress markers in the pancreatic and amygdala tissues of adolescent male rats, both with and without saffron extract treatment.

## Materials and methods

### Study design

In the current study, Wistar rats, females weighing 200 ± 30 g and males weighing 250 ± 50 g, were acquired from the Neuroscience Research Center in Tehran, Iran. They were maintained in an environment with controlled illumination (12 h light/dark cycle) and temperature (22 ± 2 °C). During the study, the animals had full access to standard food, manufactured by Behparvar Company, Tehran, Iran, and tap water. After one week of acclimatization, the rats were paired for mating overnight and separated the following morning at 11:00 am. Upon confirmation of pregnancy through vaginal smear examination for sperm, the pregnant rats were kept until they gave birth.

In this study, only male pups were included to minimize biological variability and facilitate the interpretation of the effects of saffron extract on ELS-induced metabolic and behavioral alterations. The mechanisms underlying the effects of saffron extract in this context are not yet fully understood, and fluctuations in female sex hormones across the estrous cycle are known to influence central nervous system function, HPA axis activity, stress responsiveness, and neuroendocrine regulation [20,21]. These cyclical hormonal variations may introduce additional variability that could confound the assessment of treatment effects. Therefore, male animals were selected to provide a more homogeneous experimental population and to better characterize the primary effects of saffron extract in this initial investigation. Nevertheless, the present findings may serve as a foundation for future studies examining the effects of saffron extract in female rats. We fully acknowledge the importance of including female subjects in preclinical research, particularly in studies of stress-related disorders and pharmacological interventions, and future investigations should evaluate potential sex-specific responses and the influence of the estrous cycle on treatment outcomes. To limit nutritional effects, litters were culled to 5–7 pups, and for grouping the pups, dams were randomly allocated into stress (whose pups experienced foot shock during weaning) and control (whose pups did not undergo foot shock during weaning) groups. Subsequently, the pups of each group were randomly divided into two treatment groups receiving either saline or saffron extract, resulting in four male pup subgroups: Control-Saline (CS, saline-treated), Control-Drug (CD, saffron-treated), Stress-Saline (SS, saline-treated), and Stress-Drug (SD, saffron-treated). A total of 48 male rat offspring (12 rats/group obtained from 8 litters/group) were used in the study. Among these animals, 8 rats/group (8 litters/group) were allocated to behavioral assessments as well as longitudinal body weight and food intake measurements, as this sample size is widely considered statistically appropriate for behavioral analyses in rodent studies [22]. Subsequently, 6 of these animals/group were used for fasting blood collection and pancreatic tissue sampling for biochemical and endocrine assessments [3]. However, for biochemical analyses of the amygdala, all 12 rats per group were necessary to obtain an adequate sample size. Due to the very small size of the amygdala, tissue yield from a single rat was insufficient for analysis. Therefore, tissue from at least two animals had to be combined to prepare one homogenized sample, allowing generation of 6 samples per group and requiring amygdala tissue from all 12 rats. Importantly, the experimental design involved partially overlapping subsets of animals, and the apparent differences in sample sizes reflect tissue- and procedure-specific requirements rather than separate independent cohorts. From PND11 to PND14, all pups were acclimated daily for 30 minutes in a communication box without any stress exposure. Beginning on PND15, pups in the stress groups underwent 30-minute foot shock sessions over a continuous period of five days, while control pups were placed in the box without receiving shocks. Saffron extract was administered intraperitoneally (10 mg/kg) to the CD and SD groups 30 minutes before box exposure from PND15 to PND19, while saline was given to the CS and SS groups for the same duration. The experimental procedures were carried out following the approval of the Ethics Committee of Shahid Beheshti University of Medical Sciences, Tehran, Iran (IR.SBMU.RETECH.REC.1395.13), and in accordance with the National Institutes of Health’s guidelines for laboratory animal care and use (Publication No. 80–23, revised 1996). A schematic representation of the overall experimental design and timeline, including mating, early-life stress exposure, pharmacological treatment, behavioral assessments, and tissue/blood sampling procedures, is provided in Fig 1.

**Fig 1:**
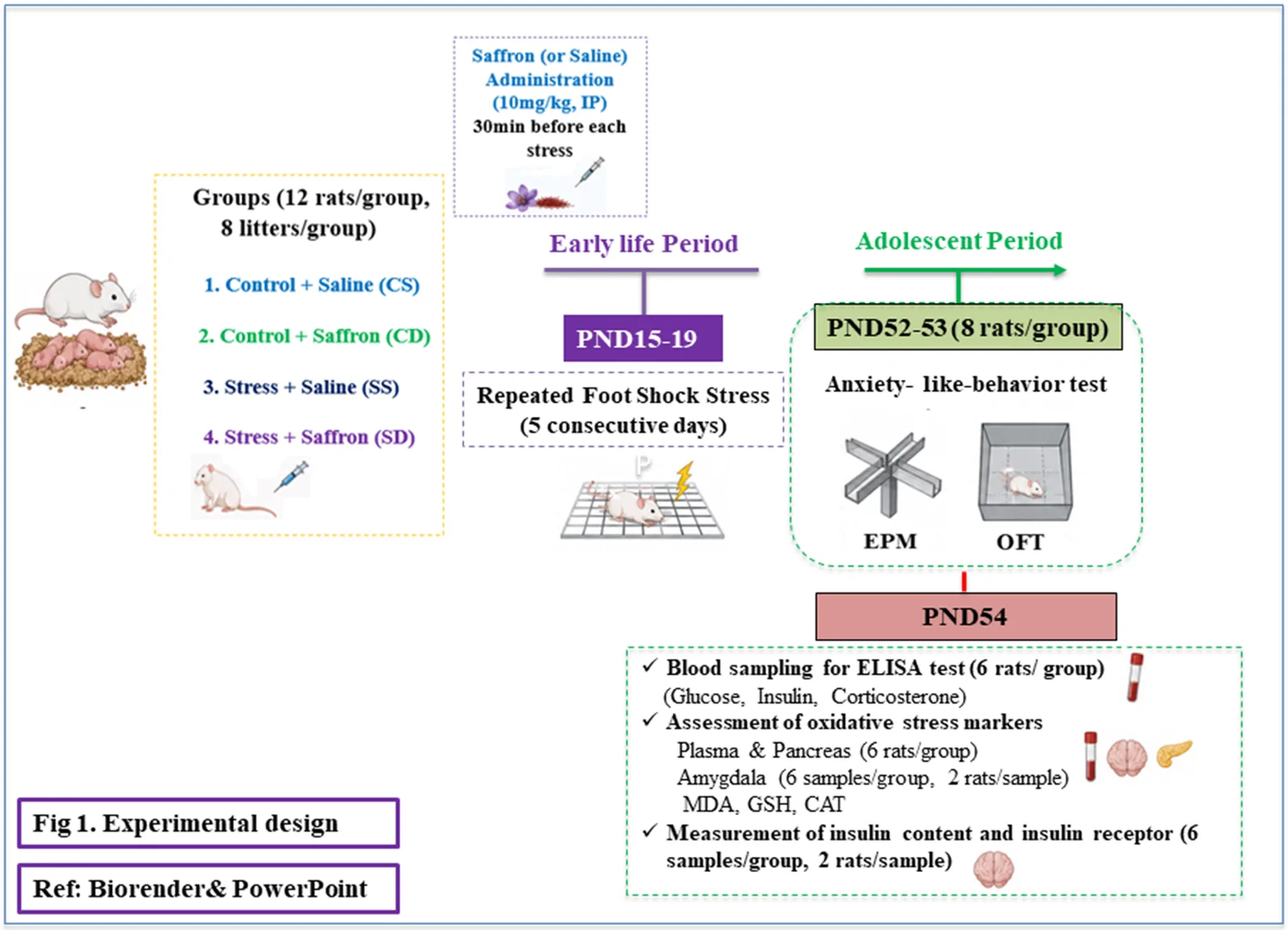
Project timeline. PND: Post Natal Day, EPM: Elevated Plus Maze, OFT: Open Field Test.

### Stress induction

Foot-shock stress was administered using a communication box with nine separate chambers. The study animals (12 rats/group, 8 litters/ group) were placed individually in each chamber and could communicate with each other through auditory, olfactory, and visual means. The floor of the communication box consists of conductive stainless steel, which is electrically connected to induce foot shock stress. Briefly, each animal was moved to the examination room one hour before the start of the experiments to allow them to adapt to the environment. Neonatal and juvenile rodents are highly sensitive to unfamiliar environments, handling, odors, and sounds [23,24]; therefore, exposure to a novel testing setting can elicit stress-related responses that may confound the effects of the foot-shock stress as a psychophysical stress. The 1-hour adaptation period allowed recovery from transportation and handling, while repeated exposure to the communication box, from PND11 to PND14, familiarized the animals with the apparatus and minimized behavioral responses attributable to environmental novelty. These procedures helped ensure that the observed responses were primarily attributable to the foot-shock stress rather than unfamiliarity with the testing environment, thereby improving the reliability and reproducibility of the behavioral assessments. From PND15 to PND19, subjects were exposed to electric shock with the frequency of 1 Hz and current of 0.8 mA, lasting 10 seconds every 60 seconds, for 30 min, once daily. The stress groups received the shock between 10:00 am and 12:00 pm, and the control groups were situated in the communication box for the same duration but without exposure to foot shock stress [25,26]. The foot-shock stress paradigm has been previously used as a psychophysical stress model for neonatal stress induction [27]. It is noteworthy that throughout the experimental period, no signs of physical injury, tissue damage, burn lesions, impaired mobility, or mortality were observed in the pups exposed to the stress procedure. Similarly, in our previous study using the same protocol, neither neonatal nor adult animals exhibited physical damage or deaths following stress exposure [26].

### Preparation of saffron extract

In this study, the red stigmas of saffron were purchased from the local market and dried. The following procedure was used to prepare the stigma extract: First, 100 g of dried stigma powder was placed in an Erlenmeyer flask (2 L) and combined with 1000 mL of distilled water and boiled for 10 minutes at 100°C (Decoction method). The resulting supernatant was filtered through a variable paper filter with a pore size of 20–30 micrometers. This solution was then placed in a bain-marie at 55°C for one day and in a sterile incubator at 33°C for one week to allow the water to evaporate slowly, leaving the extract behind. The extract was dissolved in saline and delivered through intraperitoneal injection at a dose of 10 mg/kg body weight, 30 minutes before the animals in the SD and CD groups were placed in the communication box. Meanwhile, animals in the SS and CS groups are given an intraperitoneal injection of saline, 30 minutes before being placed in the communication box [28–30].

### Ultra-performance liquid chromatography (UPLC) analysis of saffron extract

The purpose of this analysis was to examine saffron extract using Ultra Performance Liquid Chromatography (UPLC) based on a previous study [31]. The evaluation was performed using a PDA detector at three wavelengths: 250, 308, and 440 nm. Applied Knauer PLATINblue UPLC set, which includes two high-pressure pumps, an autosampler, and a PDA detector. The column used was an Eclipse-XDB-C18 with dimensions of 250 × 4.6 mm, 5 µm. The mobile phase consisted of two components: Phase A (Gradient of water) and Phase B (acetonitrile).

### Body weight and food intake measurements

Body weight was measured (8 rats/group, 8 litters/group) each day from PND15 to PND19 throughout the stress induction period, and subsequently on a weekly basis after weaning (PND26, 33, 40, 47, and 54) using a digital balance (FEW, Japan; 0.1 g resolution). Additionally, food consumption (8 rats/group, 8 litters/group) was recorded weekly from PND 27 until the end of the experiment (PND54). Animals were housed in groups (3 rats/cage) throughout the study in accordance with ethical guidelines for rat welfare. However, for weekly food intake assessment, rats were individually housed for 24 h in transparent cages positioned adjacent to one another within the animal room. Food consumption was determined by calculating the difference between the amount of food supplied and the amount remaining after the 24-h measurement period. The area under the curve (AUC) was utilized to evaluate the overall changes in each variable, including body weight and food consumption, according to the specific measurement time points established within the defined intervals.

### Blood sampling and HOMA-β index calculation

Fasting blood collection and biochemical analyses (6 rats/group, 6 litters/group) was conducted 24h following completing the behavioral tests (PND54). After a 14–16-hour fast, rats were anesthetized with isoflurane (Baxter, USA) at a dose of 6.5 mL/L/kg, adjusted according to desiccator volume and body weight. Adequate anesthesia was confirmed by the loss of reflexive responses to toe and tail pinches. Animals were subsequently euthanized by rapid decapitation using a rodent guillotine. The procedure was carried out promptly while animals remained under a surgical level of anesthesia to minimize potential distress. Trunk blood was immediately collected, between 8:00 and 8:30 am.

Blood samples were drawn into Eppendorf tubes that contained heparin (5000 IU/ml) (10 µl/ml) and centrifuged for 10 min at 664 × g. Next, the plasma was separated and stored at −80 °C for measuring the levels of corticosterone, glucose, and insulin, along with oxidative stress biomarkers [32,33].

To evaluate β-cell function, the HOMA-β index was computed using the equation below [34]:


$$\begin{array}{c} HOMA-\beta =[20\times fasting\text{\textasciitilde{}}insulin\;(\mu U/mL)/fasting\text{\textasciitilde{}}glucose\;(mM)-3.5]\text{.} \end{array}$$

### Assessment of fasting plasma glucose, insulin, and corticosterone levels

The rat insulin (Mercodia, Sweden; sensitivity: 0.07 μg/L) and corticosterone (Zellbio, Germany; sensitivity: 1.6 nmol/L) ELISA kits were utilized to measure plasma levels of insulin and corticosterone, respectively. Plasma glucose concentrations were quantified via the glucose oxidase method (Padgin Teb, Iran; sensitivity: 1 mg/dL). All analyses were conducted in a single run, with intra-assay coefficients of variation of 6.1% (insulin), 5.3% (corticosterone), and 1.8% (glucose).

### Behavioral tests

Behavioral tests were carried out on PND52 and PND53 (adolescent period) (8 rats/group, 8 litters/group). On PND52, the open field test (OFT), a standard behavioral assessment for evaluating anxiety in rats, was conducted in dim lighting (the light intensity was 15 lux) within a square chamber made of Plexiglas (60 × 60 × 50 cm). Each animal was positioned at the center of the apparatus and allowed to explore freely for 10 minutes. The floor was segmented into a central square zone measuring 30 × 30 cm and surrounding peripheral areas. A ceiling-mounted camera tracked movement, and Ethovision (Noldus, USA) analyzed parameters such as total distance travelled (for motor activity evaluation), movement velocity, as well as percentage of time spent in the central zone (CT %) (an anxiety-like behavior measure), which was calculated utilizing the formula [(Time spent in the central zone/Total time of test) ×100]. Decreased central zone time indicated greater anxiety. After each behavioral testing, animals were returned to their cages, and the apparatus was cleaned with 10% ethanol [30,35,36].

The elevated plus maze (EPM) serves as a tool for assessing anxiety-related behavior [28]. The device consists of four black Plexiglas arms positioned at right angles on a stand raised 50 cm above the ground. Each arm is 50 cm long and 10 cm wide. Two arms are open (wall-free), and 25 cm-high walls enclose the other two. A central square platform (10 x 10 cm) connects the arms at their intersection. The illumination intensity was maintained at 15 lux during this test. On PND53, the animals were placed on the maze’s central square platform toward an open arm and given 5 minutes to explore the maze voluntarily in dim, indirect light. Their behavior was recorded via an overhead ceiling-mounted camera, with data analyzed using the Ethovision (Noldus) tracking system. Measurements included the latency to first entry into the open arm, time spent in open/closed arms, total distance travelled, and the velocity of movement. The calculation of the percentage of time spent in the open arms (OAT%) was performed using the following formula:


$$\begin{array}{c} (Time\text{\textasciitilde{}}in\text{\textasciitilde{}}open\text{\textasciitilde{}}arms/[Time\text{\textasciitilde{}}in\text{\textasciitilde{}}open\text{\textasciitilde{}}arms+Time\text{\textasciitilde{}}in\text{\textasciitilde{}}closed\text{\textasciitilde{}}arms])\times 100. \end{array}$$

A significant decrease in the percentage of time spent in the open arms (OAT%) during the elevated plus maze test was regarded as a sign of anxiety-like behavior.

### Pancreatic tissue isolation and preparation

Twenty-four hours after completing behavioral testing (PND54), following decapitation and blood collection, as described in detail above, pancreatic tissue was removed (6 rats/group, 6 litters/group). The isolated pancreas was flash-frozen in liquid nitrogen for 60 seconds and stored at −80°C for subsequent assays.

For the assessments, the frozen pancreas was carefully dissected into smaller pieces and homogenized in lysis buffer [Tris-HCl pH 8, sodium deoxycholate, NaCl, SDS, EDTA, Triton, and H_2_O] using a laboratory homogenizer (TOMY Micro Smash MS 100, Indonesia). After centrifugation (12,000 rpm, 30 min), the clear supernatant was collected for total protein determination via the Bradford method and subsequent evaluations [2].

### Amygdala tissue isolation and preparation

To isolate the amygdala, following decapitation as described above, brain tissues were collected from all experimental animals (12 rats/group, 8 litters/group). Following decapitation as described above, the rat’s skull was carefully opened using scissors, and bone sheaths were removed with caution to avoid damaging the brain tissue. All steps of the isolation procedure were performed on crushed ice. The brain was extracted with a flat spatula, and the hippocampus was identified. Using a brain matrix, the brain was coronally sectioned anterior to and posterior to the hippocampus. The remaining anterior and posterior brain sections were discarded, and the brain was flipped onto its frontal end. Subsequently, the amygdala from both hemispheres were extracted, rapidly frozen in liquid nitrogen for 60 seconds, and kept at −80°C until further testing [37]. For the assessments, the frozen amygdala 2 amygdala per sample, 6 samples/group) was homogenized in lysis buffer [Tris-HCl pH 8, sodium deoxycholate, NaCl, SDS, EDTA, Triton, and H_2_O] using a laboratory homogenizer (TOMY Micro Smash MS 100, Indonesia). After centrifugation (12,000 rpm, 30 min), the supernatant was collected to measure total protein concentration (using the Bradford assay) and subsequent assessments (Amygdala oxidative stress markers, amygdala insulin content and insulin receptor protein level) [2].

### Determination of amygdala insulin content and insulin receptor protein level

The prepared amygdala supernatant was utilized to determine the protein level of insulin receptor and the insulin content, using a rat insulin receptor ELISA kit (minimum detection: 0.2 ng/ml; Zellbio, Germany) and an ultra-sensitive rat insulin ELISA kit (minimum detection: 0.02 μg/l; Mercodia, Sweden), respectively. The measurements were performed in one run, and the intra-assay coefficients of variation were 5.9% and 6.1%, respectively.

### Assessment of malondialdehyde (MDA) and glutathione (GSH) levels, and catalase activity in the Pancreas, Amygdala, and Plasma

The level of MDA, a marker of lipid peroxidation, in the plasma and the supernatant of pancreas and amygdala was determined using a commercial colorimetric assay kit (Zellbio, Germany, sensitivity 0.1 μM). The levels of this oxidative stress indicator were reported relative to the amount of protein (μmol/mg protein) or μmol/ml for plasma [38].

The Ellman method was employed to measure GSH levels in the pancreas, amygdala, and plasma. Briefly, phosphate-buffered saline (PBS) was mixed with pancreatic homogenate or plasma (containing 120 μg protein) and amygdala homogenate (90 μg protein). Trichloroacetic acid was then added, and the mixture was centrifuged. Incubation of the supernatant with DTNB [5,5-dithiobis-2-nitrobenzoic acid (2 mg/mL in PBS)] (D218200, Sigma, USA) was carried out at 37°C for 10 minutes, followed by absorbance measurement at 405 nm on an ELISA reader (BioTek, ELX800TS, USA). GSH content was reported in μmol/mg protein or μmol/ml for plasma [39,40].

Using the Goth method, catalase activity was assessed. For this procedure, PBS was added to the plasma (180 μg protein), the pancreatic supernatant (180 μg protein), and the amygdala supernatant (120 μg protein). Each of the mixtures was then incubated with 0.01 M hydrogen peroxide (H_2_O_2_) at 25°C for 15 minutes. The enzymatic reaction was halted by adding ammonium molybdate (6.35 mg/mL in PBS) (277908, Sigma, USA). The breakdown rate of H_2_O_2_ was assessed by measuring absorbance at 405 nm, with results expressed as micromoles of H_2_O_2_ degraded per minute per milligram of protein (μmol H_2_O_2_/min/mg protein) or mU/min/ml for plasma [41–43].

### Statistical analysis

Sample sizes were determined based on our previous studies employing similar behavioral, endocrine, and oxidative stress paradigms, in which these group sizes were sufficient to detect statistically significant differences among experimental groups [3, 22]. All data are presented as mean ± SEM. Two-way analysis of variance (ANOVA) (with stress and drug as independent factors) and three-way mixed ANOVA (with time as the within-subject factor and stress and drug as independent factors) were performed, followed by Tukey’s multiple-comparisons post hoc test, using GraphPad Prism 9 and SPSS Statistics 21. Pearson correlation analysis was also performed using R software to evaluate the associations between anxiety-related and locomotor activity parameters. P values < 0.05 were considered statistically significant.

## Results

### Ultra-performance liquid chromatography (UPLC) analysis of saffron extract

A UPLC analysis of saffron extract revealed peaks indicative of bioactive compounds (Fig 2). The evaluation was performed at three wavelengths: 250, 308, and 440 nm. Six components of the saffron extract were characterized and identified along with their retention times (RT) as shown in Table 1.

**Table 1:** The specification of identified components of the saffron extract by UPLC.

Compound	Retention time (RT) (min)	λmax (nm)
Picrocrocin	7.8	250
Trans-crocin I	19.4	440
Trans-crocin II	22.3	440
Cis-crocin I	26.3	440
Cis-crocin II	30.6	440
Safranal	43.4	308

**Fig 2:**
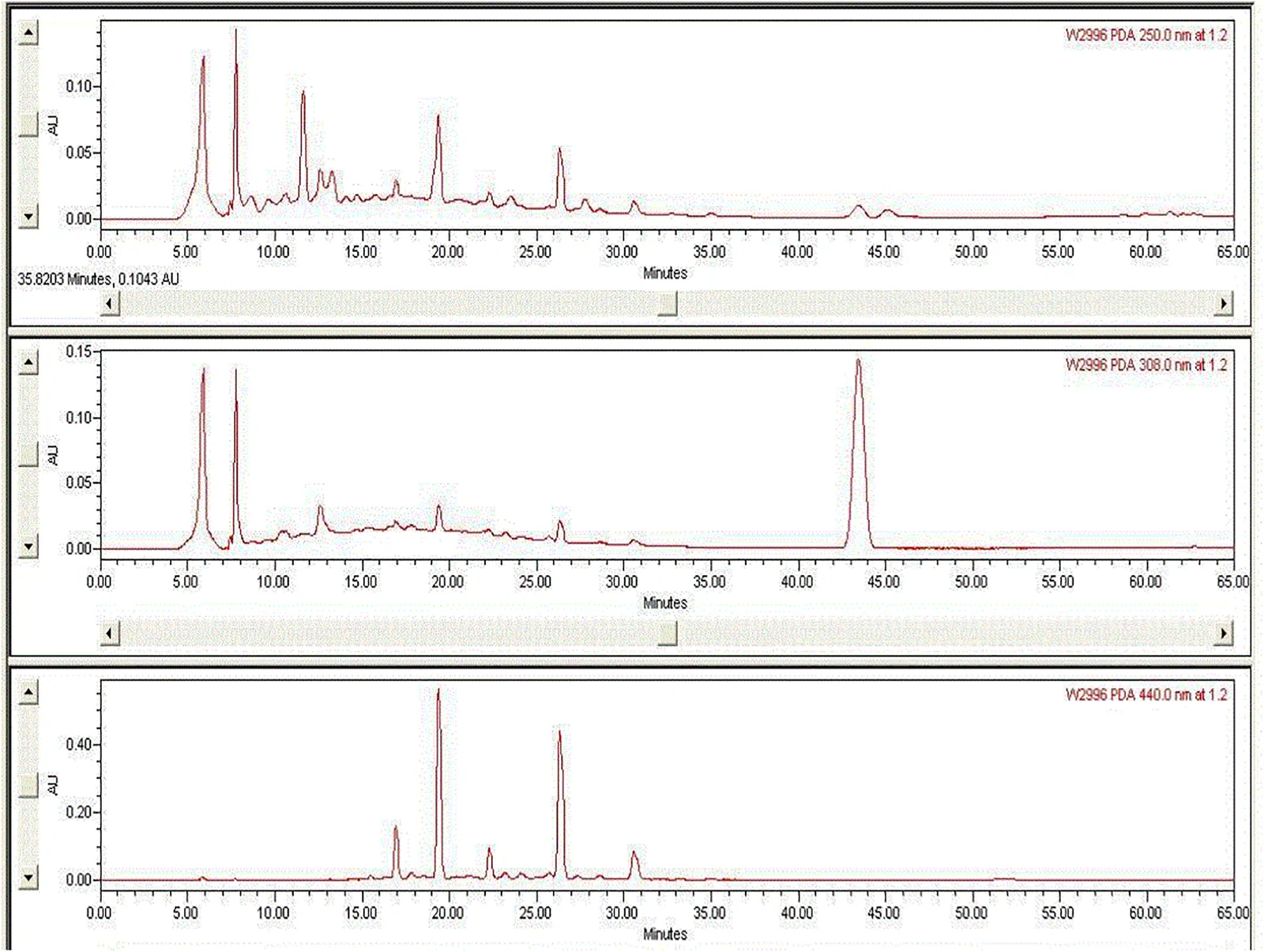
Chromatogram of saffron extract (250,308, 440nm).

### The effect of ELS and/or saffron extract on plasma corticosterone concentration in adolescent male rats

A two‑way ANOVA was performed to evaluate the effects of stress and drug on plasma corticosterone concentration. There was a significant effect of stress (F (1, 20) = 273.5, P < 0.0001) and drug (F (1, 20) = 208.3, P < 0.0001), as well as a significant interaction between stress and drug (F (1, 20) = 115.3, P < 0.0001). Tukey’s multiple comparison test indicated that plasma corticosterone concentration was markedly higher in the SS group than in the CS group (P < 0.0001; Fig 3). The administration of saffron extract in the SD group significantly reduced corticosterone levels compared to the SS group (P < 0.0001, Fig 3); therefore, no significant difference was observed compared to the control groups (Fig 3). Saffron extract administration in the CD group did not change this plasma parameter compared to the CS group (Fig 3).

**Fig 3:**
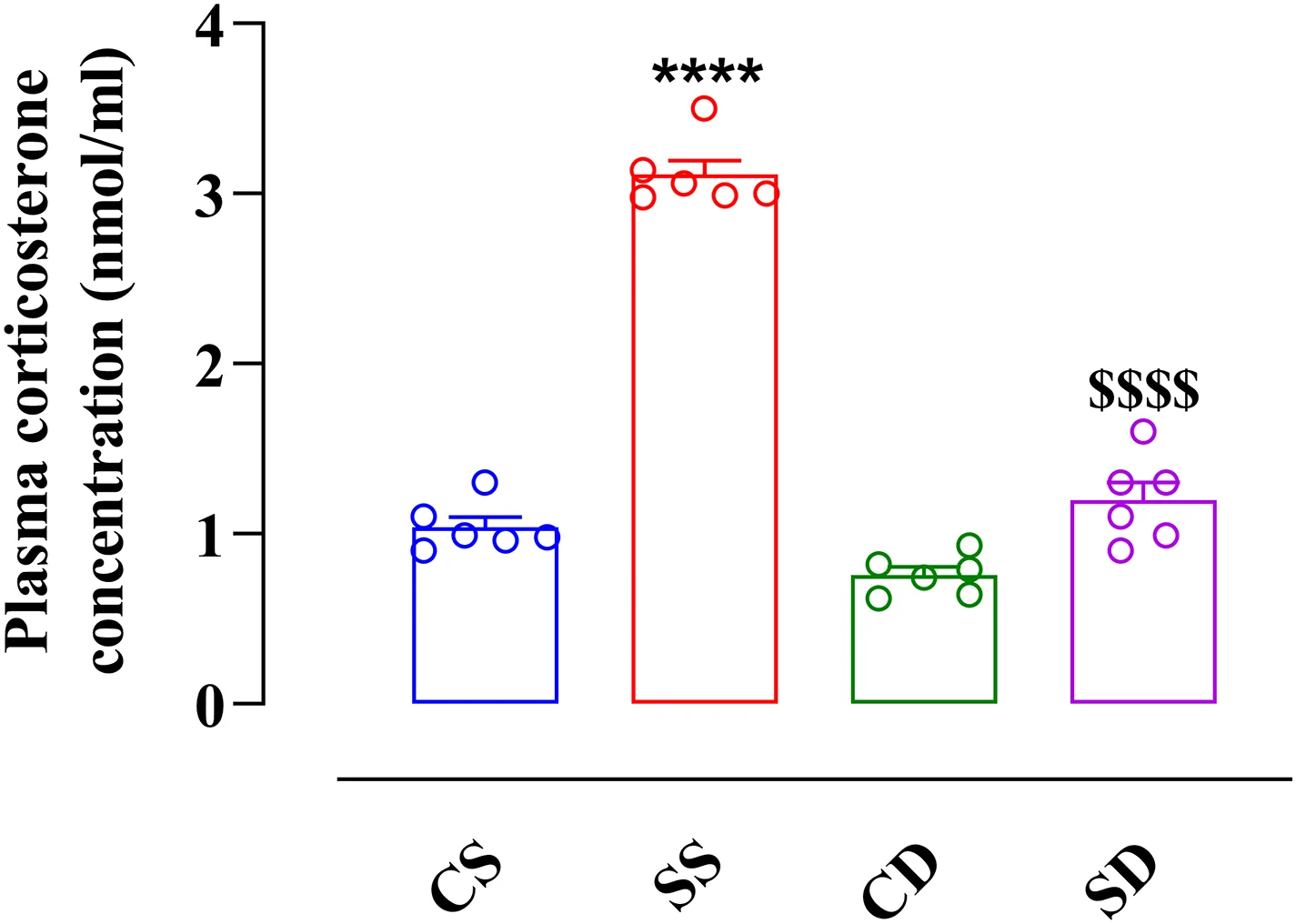
Effect of early life stress and/or saffron extract on plasma corticosterone concentration in adolescent male rats. Each column represents the mean ±SEM (6 rats/group). ^****^p < 0.0001 versus Control+ Saline group, ^$$$$^p < 0.0001versus Stress+ Saline group. **S**: Saline, **D**: Drug (saffron extract).

### The effect of ELS and/or saffron extract on body weight and food intake in adolescent male rats

Body weight and food intake of animals did not show any significant difference between the experimental groups at any time point as shown by Tukey’s multiple comparison test followed by three-way mixed ANOVA (Fig 4A, 4B). Regarding body weight changes, the three-way mixed ANOVA revealed the following F statistics for the main effects and their interactions: stress (F (1, 28) = 0.571, P = 0.456), drug (F (1, 28) = 0.011, P = 0.918), stress × drug interaction (F (1, 28) = 0.366, P = 0.550), time (F (1.417, 39.68) = 5534, P < 0.0001), time × stress interaction (F (9, 252) = 0.920, P = 0.508), time × drug interaction (F (9, 252) = 0.943, P = 0.488), and time × stress × drug interaction (F (9, 252) = 0.884, P = 0.540). Regarding changes in food intake, the three-way mixed ANOVA yielded the following F statistics for the main effects and their interactions: stress (F (1, 28) = 5.239, P = 0.030), drug (F (1, 28) = 0.107, P = 0.746), stress × drug interaction (F (1, 28) = 0.048, P = 0.829), time (F (2.529, 70.82) = 88.930, P < 0.0001), time × stress interaction (F (4, 112) = 2.364, P = 0.057), time × drug interaction (F (4, 112) = 1.479, P = 0.213), and time × stress × drug interaction (F (4, 112) = 0.682, P = 0.606).

**Fig 4:**
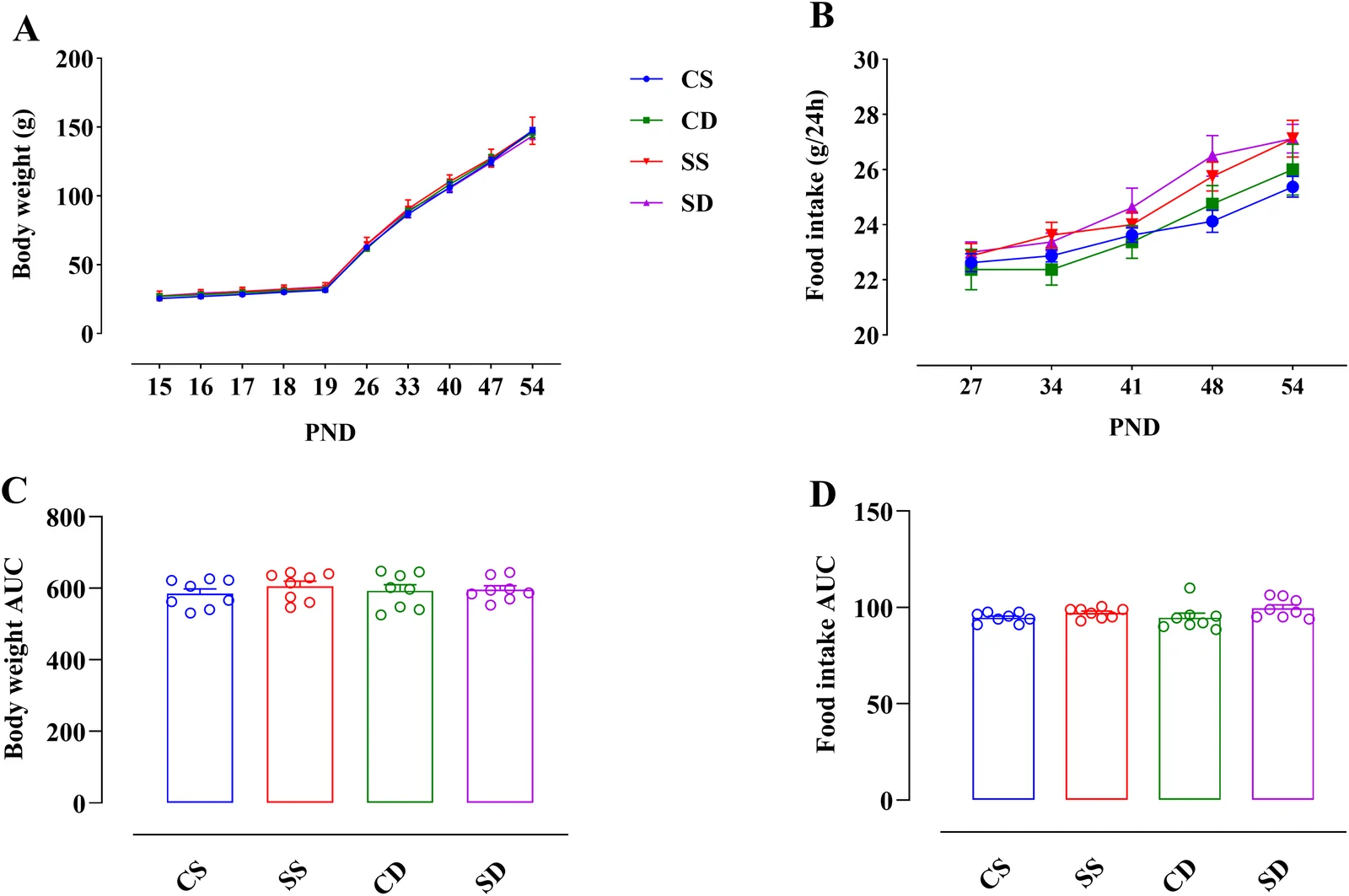
Effect of early life stress and/or saffron extract on body weight (A), food intake (B), body weight AUC (C), and food intake AUC (D) in adolescent male rats. Each point or column represents the mean±SEM (8 rats/group). **CS:** Control+ Saline**, CD:** Control+Drug **(**saffron extract**), SS:** Stress+ Saline, **SD**: Stress+Drug (saffron extract). **S**: Saline, **D**: Drug (saffron extract).

Two‑way ANOVA analysis for body weight AUC (Fig 4C) did not show any significant effect of stress (F (1, 28) = 0.749, P = 0.394) and drug (F (1, 28) = 0.002, P = 0.964), as well as interaction between stress and drug (F (1, 28) = 0.387, P = 0.539). Two‑way ANOVA analysis for food intake AUC (Fig 4D) showed a significant effect of stress (F (1, 28) = 5.758, P = 0.023) and a non-significant effect of drug (F (1, 28) = 0.121, P = 0.731), as well as an interaction between stress and drug (F (1, 28) = 0.098, P = 0.757). Tukey’s multiple comparison test indicated that there were no notable differences in the AUC for body weight and food intake among the experimental groups (Fig 4C, 4D).

### The effect of ELS and/or saffron extract on fasting plasma glucose and insulin levels, and Homeostasis Model Assessment of β-cell function (HOMA-β) index in adolescent male rats

To analyse the effects of stress and drug on plasma glucose concentration a two‑way ANOVA was and showed a significant effect of stress (F (1, 20) = 59.28, P < 0.0001) and drug (F (1, 20) = 49.26, P < 0.0001), as well as stress and drug interaction (F (1, 20) = 30.83, P < 0.0001). Tukey’s multiple comparison test indicated that fasting plasma glucose concentration was significantly higher in the SS group than in the CS group due to ELS (P < 0.0001, Fig 5A). Injection of saffron extract in the SD group significantly brought down the fasting plasma glucose level in comparison to the SS group (P < 0.0001, Fig 5A).

**Fig 5:**
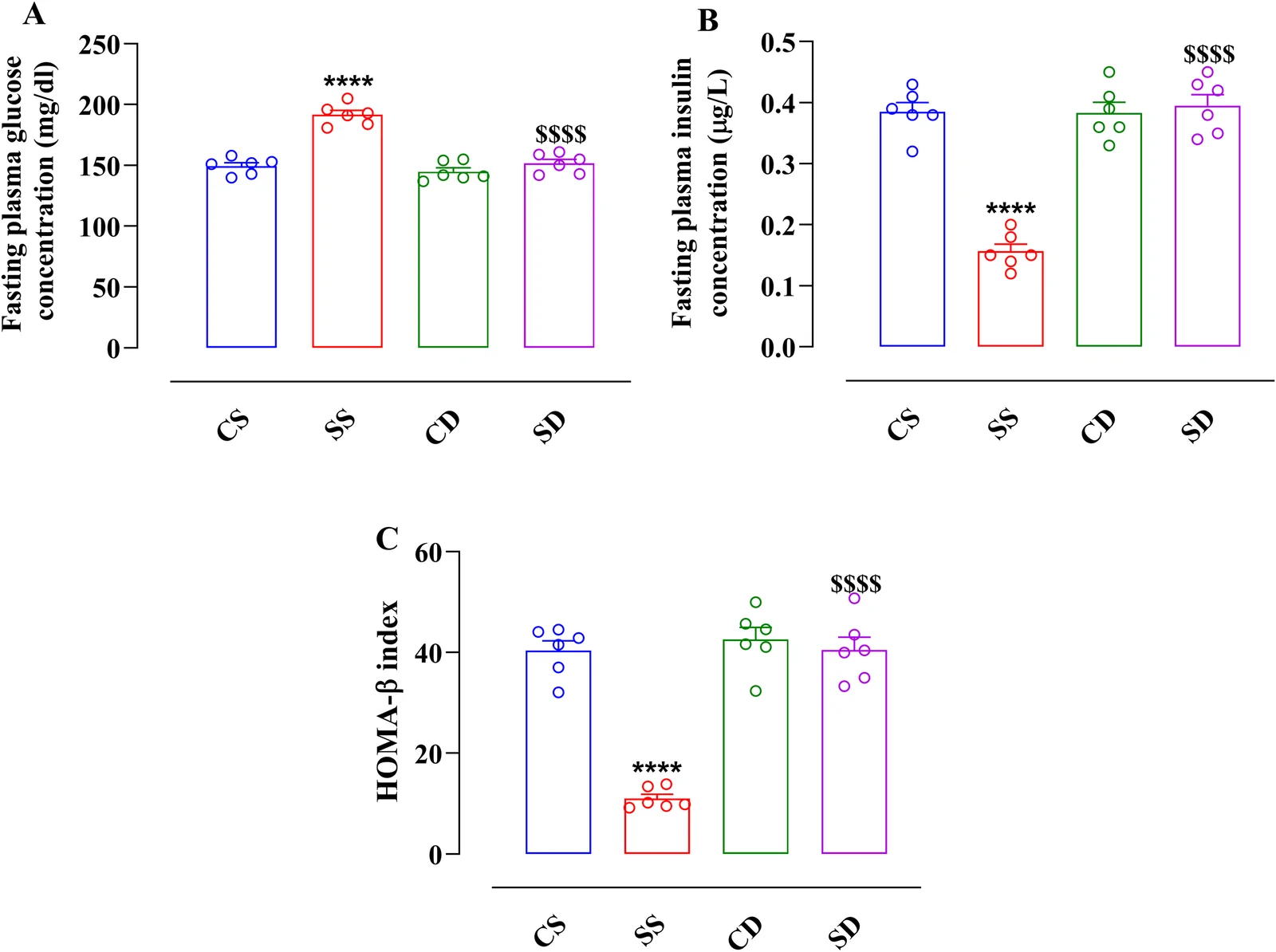
Effect of early life stress and/or saffron extract on fasting plasma (A) glucose and (B) insulin concentrations, and (C) HOMA-β index in adolescent male rats. Each column represents the mean±SEM (6 rats/group). ^****^p < 0.0001 versus Control+ Saline group, ^$$$$^p < 0.0001versus Stress+ Saline group. **S**: Saline, **D**: Drug (saffron extract), HOMA-β: Homeostasis Model Assessment of β-cell function.

The effects of stress and drugs on plasma insulin concentration and HOMA-β index were also analysed by a two-way ANOVA. The results showed a significant effect of stress (F (1, 20) = 46.38, P < 0.0001) and drug (F (1, 20) = 55.33, P < 0.0001), as well as a significant interaction between stress and drug (F (1, 20) = 56.90, P < 0.0001) on insulin concentration. Also, there was a significant effect of stress (F (1, 20) = 57.76, P < 0.0001) and drug (F (1, 20) = 58.83, P < 0.0001), as well as a significant interaction between stress and drug (F (1, 20) = 43.56, P < 0.0001) on the HOMA-β index. According to Tukey’s multiple comparison test, ELS caused a significant reduction in fasting plasma insulin and HOMA-β index in the SS group relative to the CS group (P < 0.0001, Fig 5B, 5C). Saffron extract could significantly increase fasting plasma insulin level and HOMA-β index in the SD group compared to the SS group (P < 0.0001, Fig 5B, 5C). However, the administration of saffron extract in the CD group did not markedly affect the mentioned parameters relative to the CS group (Fig 5A–5C).

### The effect of ELS and/or saffron extract on plasma, pancreatic, and amygdala MDA and GSH levels, as well as catalase activity in adolescent male rats

Two-way ANOVA indicated a significant effect of stress (F (1, 20) = 47.83, P < 0.0001) and drug (F (1, 20) = 31.36, P < 0.0001), as well as stress and drug interaction (F (1, 20) = 44.82, P < 0.0001) on plasma MDA level. Moreover, there was a significant effect of stress (F (1, 20) = 26.17, P < 0.0001), drug (F (1, 20) = 31.57, P < 0.0001), and the interaction of stress and drug (F (1, 20) = 18.29, P = 0.0004) on plasma GSH content. Tukey’s multiple comparison test indicated that ELS induction led to a significant increase in plasma MDA level (P < 0.0001, Fig 6A) and a significant decrease in plasma GSH content in the SS group compared to the CS group (P < 0.0001, Fig 6B). Injection of saffron extract in the SD group lowered MDA levels and raised GSH levels relative to the SS group (P < 0.0001), whereas saffron treatment did not alter these parameters in the CD group (Fig 6A, 6B). Additionally, no significant differences were found when comparing the SD group with the control groups (Fig 6A, 6B).

**Fig 6:**
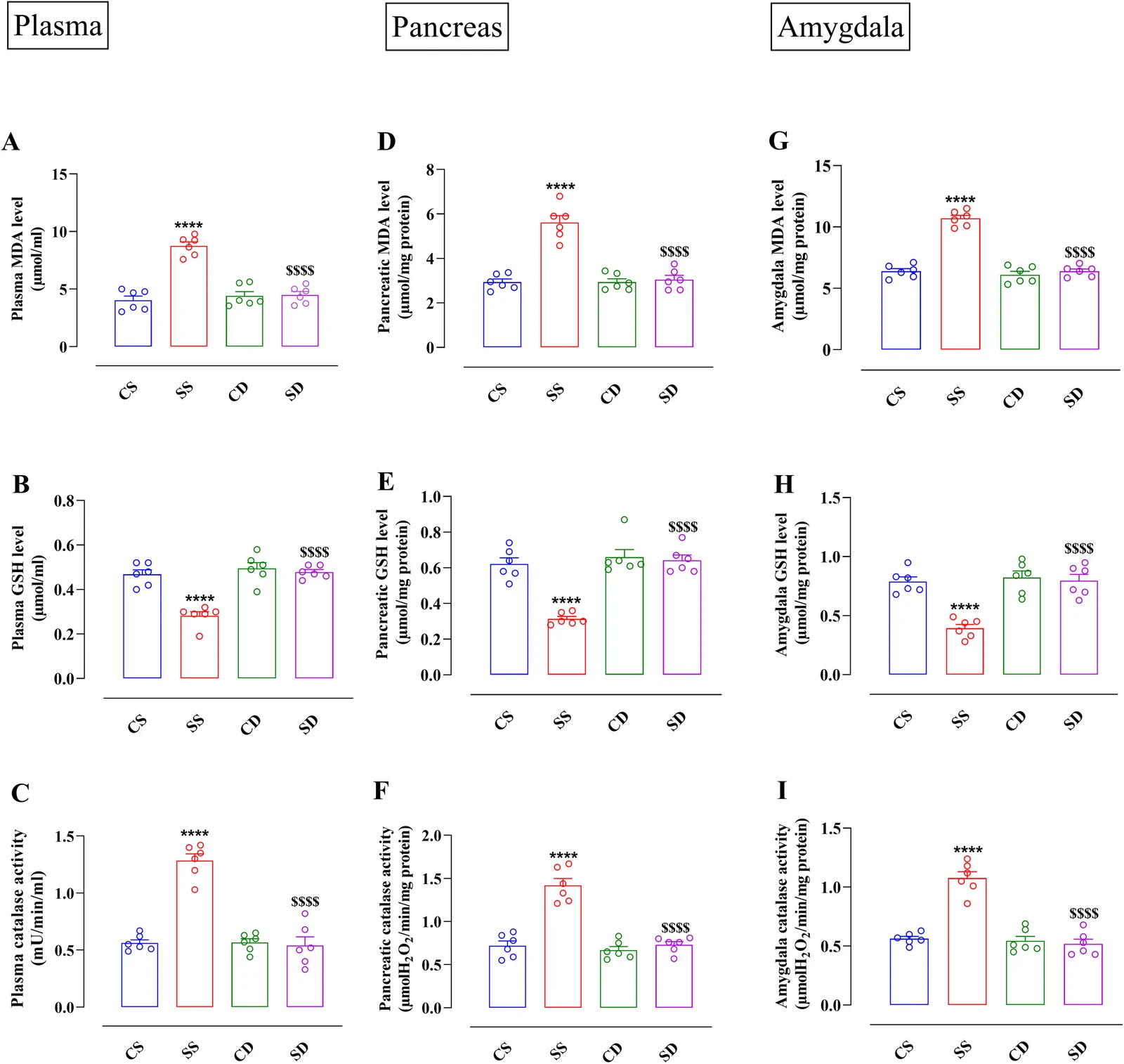
Effect of early life stress and/or saffron extract on plasma [(A) MDA and (B) GSH levels and (C) catalase activity], pancreatic [(D) MDA and (E) GSH levels and (F) catalase activity], and amygdala [(G) MDA and (H) GSH levels and (I) catalase activity], in adolescent male rats. Each column represents the mean±SEM (6 rats/group). ^****^p < 0.0001 versus Control+ Saline group, ^$$$$^p < 0.0001versus Stress+ Saline group. **S**: Saline, **D**: Drug (saffron extract).

The same statistical analysis showed a significant effect of stress (F (1, 20) = 44.50, P < 0.0001) and drug (F (1, 20) = 49.75, P < 0.0001), as well as stress and drug interaction (F (1, 20) = 51.11, P < 0.0001) on plasma catalase activity. Tukey’s multiple comparison test indicated that following stress induction, plasma catalase activity was elevated in the SS group compared to the CS group (P < 0.0001, Fig 6C). Saffron extract administration reduced catalase activity in the SD group compared to the SS group (P < 0.0001), but did not influence catalase activity in the CD group (Fig 6C). There was no statistically significant difference between the SD group and the control group (Fig 6C).

Considering two‑way ANOVA statistical analysis, there was a significant effect of stress (F (1, 20) = 44.39, P < 0.0001) and drug (F (1, 20) = 37.38, P < 0.0001), as well as a significant interaction between stress and drug (F (1, 20) = 37.47, P < 0.0001) on pancreatic MDA level. Moreover, there was a significant effect of stress (F (1, 20) = 26.13, P < 0.0001) and drug (F (1, 20) = 32.93, P < 0.0001), as well as a significant interaction between stress and drug (F (1, 20) = 20.60, P = 0.0002) on pancreatic GSH level. Tukey’s multiple comparison test indicated that in the SS group, ELS caused a notable rise in the pancreatic MDA level alongside a decrease in GSH content relative to the CS group (P < 0.0001) (Fig 6D, 6E). The test showed that in the SD group, administration of saffron extract markedly raised pancreatic GSH content and lowered MDA concentration relative to the SS group (P < 0.0001). However, administration of this extract did not affect these parameters in the CD group (Fig 6D, 6E). There was no significant difference between the SD group and the CS and CD groups (Fig 6D, 6E).

In accordance with two‑way ANOVA, there was a significant effect of stress (F (1, 20) = 46.25, P < 0.0001), drug (F (1, 20) = 43.86, P < 0.0001), and the interaction between stress and drug (F (1, 20) = 32.49, P < 0.0001) on pancreatic catalase activity. Tukey’s multiple comparison test indicated that after exposure to the ELS, the pancreatic catalase activity in the SS group showed a substantial increase in comparison to CS group (P < 0.0001, Fig 6F). In the SD group, activity of this enzyme was markedly lowered following saffron extract injection in comparison to the SS group (P < 0.0001); however, administration of this extract did not affect catalase activity in the CD group (Fig 6F). The SD group did not exhibit any differences compared to the CS and CD groups (Fig 6F).

Based on a two-way ANOVA test, there was a significant effect of stress (F (1, 20) = 96.88, P < 0.0001) and drug (F (1, 20) = 96.04, P < 0.0001), as well as a significant interaction between stress and drug (F (1, 20) = 73.52, P < 0.0001) on amygdala MDA level. Also, there was a significant effect of stress (F (1, 20) = 21.01, P = 0.0002), drug (F (1, 20) = 22.70, P = 0.0001), as well as stress and drug interaction (F (1, 20) = 16.03, P = 0.0007) on amygdala GSH level. According to Tukey’s multiple comparison test, the amygdala MDA level (P < 0.0001, Fig 6G) was markedly increased, and the GSH level (P < 0.0001, Fig 6H) was notably decreased in the SS group following early life stress induction. Injection of saffron extract meaningfully reduced MDA level and increased its GSH level in the SD group (P < 0.0001, Fig 6G, 6H). There was no significant difference between the SD group and the CS and CD groups (Fig 6G, 6H).

As reported by two‑way ANOVA, a significant effect of stress (F (1, 20) = 35.79, P < 0.0001) and drug (F (1, 20) = 50.20, P < 0.0001), as well as stress and drug interaction (F (1, 20) = 43.50, P < 0.0001), was observed on amygdala catalase activity. In this regard, Tukey’s multiple comparison test indicated that amygdala catalase activity significantly increased in SS group following stress induction in early life (P > 0.0001, Fig 6I). In SD group after saffron extract infusion, the activity of this enzyme significantly decreased compared to SS group (P < 0.0001, Fig 6I). The SD group showed no statistically meaningful variation when compared to the control groups (Fig 6I).

### The effect of ELS and/or saffron extract on amygdala insulin content and insulin receptor protein level in adolescent male rats

Based on the results of the two‑way ANOVA, a significant effect of stress (F (1, 20) = 42.14, P < 0.0001) and drug (F (1, 20) = 45.52, P < 0.0001), as well as a significant interaction between stress and drug (F (1, 20) = 29.78, P < 0.0001) was detected on amygdala insulin content. Moreover, there was a significant effect of stress (F (1, 20) = 32.11, P < 0.0001), drug (F (1, 20) = 22.84, P = 0.0001), along with stress and drug interaction (F (1, 20) = 24.56, P < 0.0001) on amygdala insulin receptor protein level. Tukey’s multiple comparison test reported that the amygdala insulin content decreased (P < 0.0001, Fig 7A) and insulin receptor protein level increased significantly (P < 0.0001, Fig 7B) in the SS group compared to the CS group (Fig 7A, 7B). Treatment with saffron extract led to a significant increase in amygdala insulin content (P < 0.0001, Fig 7A) and a significant decrease in amygdala insulin receptor protein levels in the SD group compared to the SS group (P < 0.0001, Fig 7B). Conversely, injection of saffron extract in the CD group did not influence either the insulin content or insulin receptor levels in the amygdala. No statistically significant differences were observed between the SD group and the control groups (Fig 7A, 7B).

**Fig 7:**
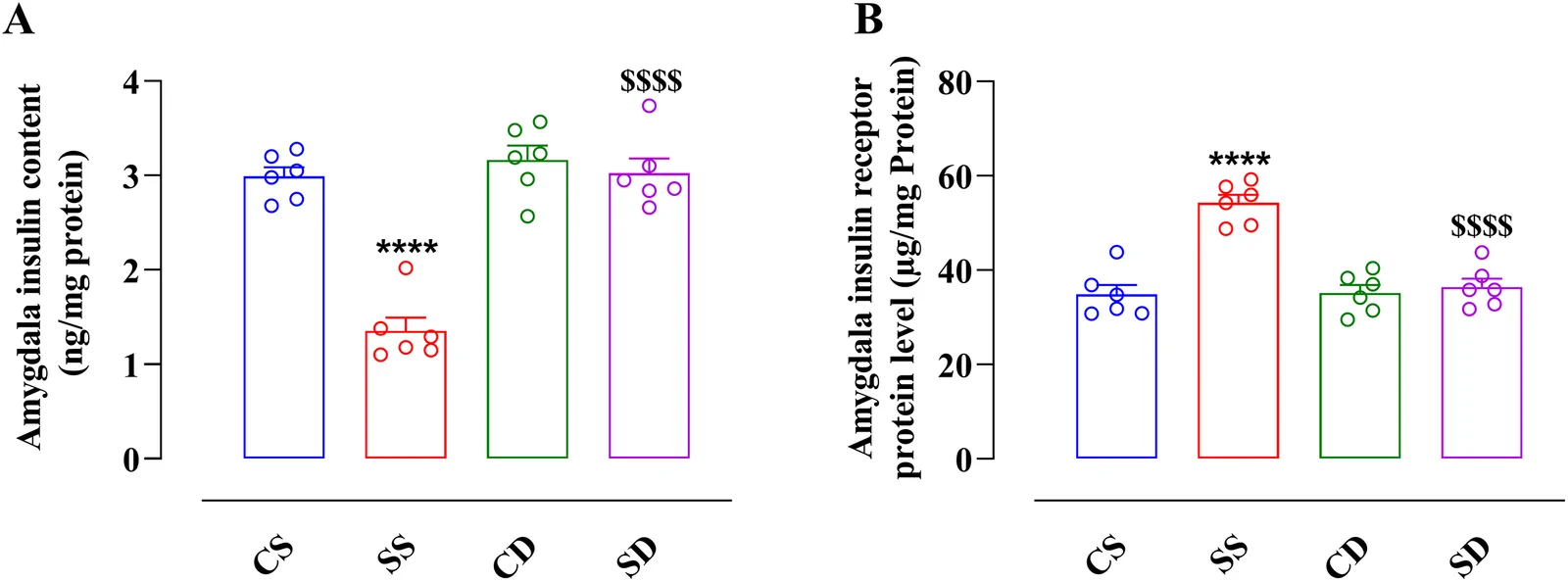
Effect of early life stress and/or saffron extract on amygdala (A) insulin content and (B) insulin receptor protein level in adolescent male rats. Each column represents the mean±SEM (6 samples/group, 2 rats/sample). ^****^p < 0.0001 versus Control+ Saline group, ^$$$$^p < 0.0001versus Stress+ Saline group. **S**: Saline, **D**: Drug (saffron extract).

### The effect of ELS and/or saffron extract on the parameters measured following Open Field Test (OFT) in adolescent male rats

A two‑way ANOVA was utilized to assess the effects of stress and drug on the OFT results. In detail, there was a significant effect of stress (F (1, 28) = 103.2, P < 0.0001), (F (1, 28) = 10.96, P = 0.0026) and drug (F (1, 28) = 64.91, P < 0.0001), (F (1, 28) = 17.49, P = 0.0003), as well as stress and drug interaction (F (1, 28) = 74.30, P < 0.0001), (F (1, 28) = 22.12, P < 0.0001) on total distance traveled and movement velocity, respectively. Moreover, there was a significant effect of stress (F (1, 28) = 45.43, P < 0.0001) and drug (F (1, 28) = 42.76, P < 0.0001), as well as stress and drug interaction (F (1, 28) = 53.14, P < 0.0001) on the percentage of time spent in the central zone (CT%). Tukey’s multiple comparison test of OFT indicated that locomotor activity and anxiety-related behavior were increased in the SS group compared to the CS group. In detail, the total distance traveled and movement velocity (P < 0.0001) (Fig 8A, 8B) were significantly increased, and CT% (P < 0.0001, Fig 8C) was markedly decreased in the SS group compared to the CS group. In the SD group, administration of saffron extract resulted in a significant decrease in the total distance traveled and movement velocity (P < 0.0001, Fig 8A, 8B) and a considerable increase in CT% (P < 0.0001, Fig 8C) compared to the SS group. In this respect, no statistically meaningful variation was detected in the SD group relative to the control groups (Fig 8A–8C).

**Fig 8:**
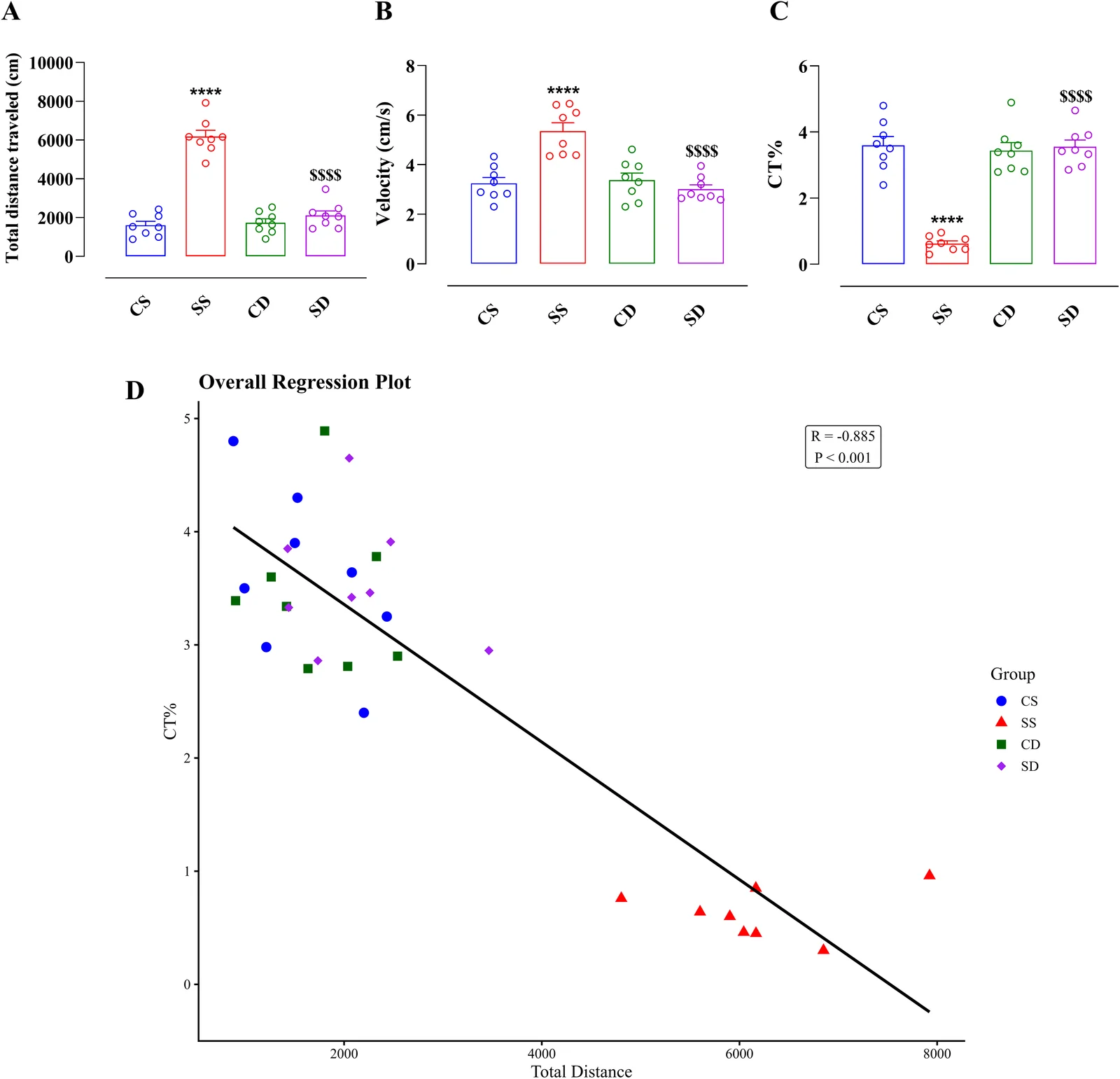
Effect of early life stress and/or saffron extract on (A) total distance travelled, (B) velocity, (C) CT%, and (D) the correlation between total distance travelled and CT% obtained from OFT in adolescent male rats. Each column represents the mean±SEM (8 rats/group). ^****^p < 0.0001 versus Control+Saline group, ^$$$$^p < 0.0001versus Stress+Saline group. Correlation analysis across all experimental groups (CS, SS, CD, and SD) revealed a strong negative correlation between total distance travelled and CT%. **S**: Saline, **D**: Drug (saffron extract)**, CT%**: percentage of time spent in the center zone, **OFT**: Open Field Test.

The correlation analysis across all study groups (CS, SS, CD, and SD) revealed a strong negative correlation between total distance traveled and CT% obtained from the OFT (Pearson’s coefficient = −0.885, P < 0.001) (Fig 8D).

### The effect of ELS and/or saffron extract on the parameters measured following Elevated Plus Maze (EPM) testing in adolescent male rats

In EPM test, two‑way ANOVA showed a significant effect of stress (F (1, 28) = 462.8, P < 0.0001), (F (1, 28) = 46.88, P < 0.0001), (F (1, 28) = 46.37, P < 0.0001) and drug (F (1, 28) = 445.0, P < 0.0001), (F (1, 28) = 47.70, P < 0.0001), (F (1, 28) = 33.91, P < 0.0001), as well as stress and drug interaction (F (1, 28) = 450.1, P < 0.0001), (F (1, 28) = 45.88, P < 0.0001),), (F (1, 28) = 40.96, P < 0.0001) on latency to first entry into the open arm, total distance traveled, and velocity of movement, respectively. Also, there was a significant effect of stress (F (1, 28) = 9.400, P = 0.0048) and drug (F (1, 28) = 23.63, P < 0.0001), as well as a significant interaction between stress and drug (F (1, 28) = 17.61, P = 0.0002) on the percentage of time spent in the open arms (OAT%). Tukey’s multiple comparison test indicated that following the induction of ELS, there was a substantial increase in latency to first entry into the open arm, total distance traveled, and velocity of movement (P < 0.0001, Fig 9A–9C), while OAT% showed a remarkable reduction (P < 0.0001) in SS group compared to CS group (Fig 9D). Injection of saffron extract led to a significant decline in the latency to first entry into the open arm, total distance traveled, and velocity of movement (P < 0.0001) (Fig 9A–9C) along with a marked rise in OAT% in SD group (P < 0.0001, Fig 9D) in comparison to the SS group. In contrast, saffron extract administration in the CD group did not affect the mentioned parameters. Moreover, no statistically notable difference was observed between the SD group and the control groups (Fig 9A–9D).

**Fig 9:**
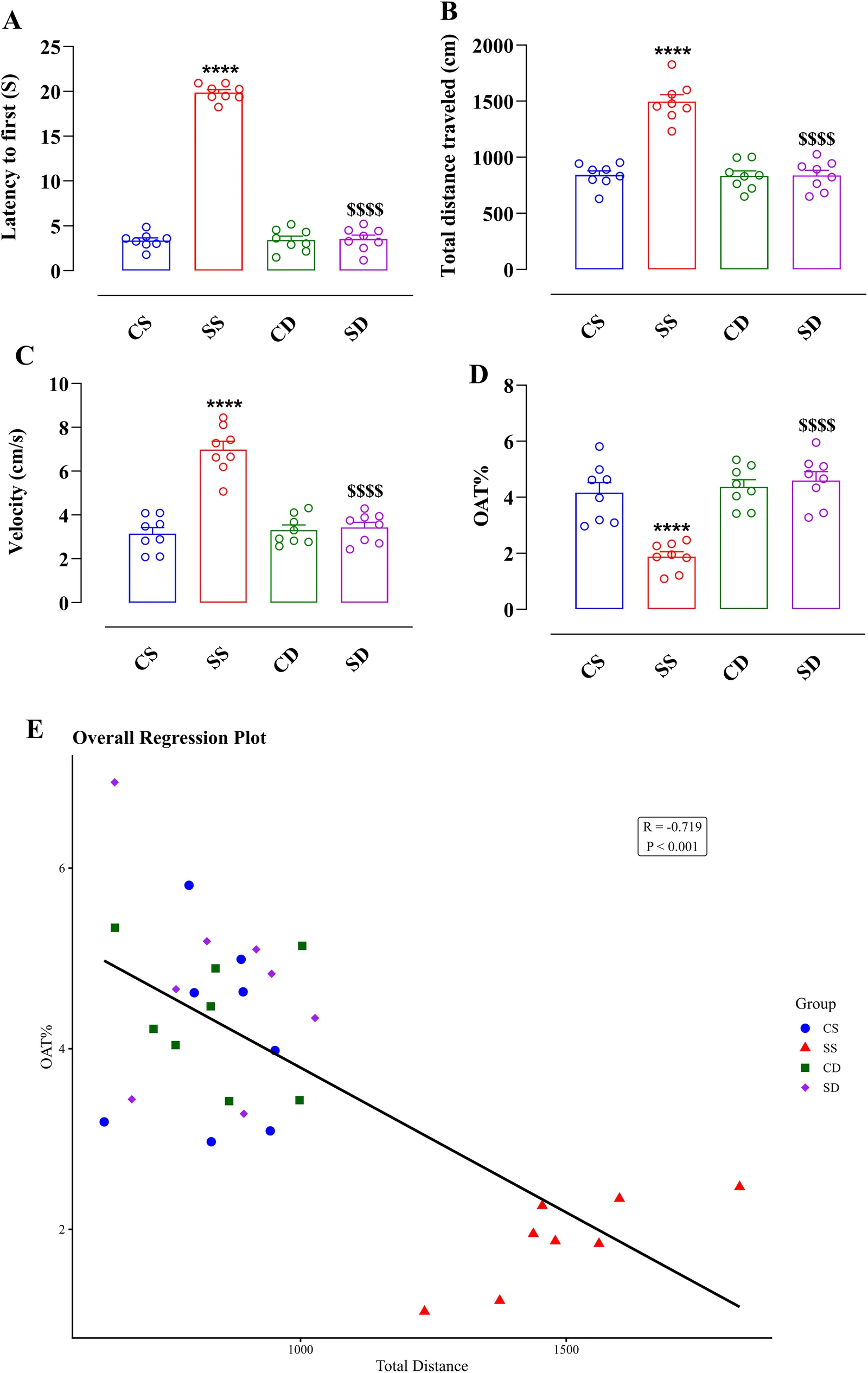
Effect of early life stress and/or saffron extract on (A) latency to first, (B) total distance travelled, (C) velocity, and (D) OAT%, and (E) the correlation between total distance travelled and OAT% obtained from EPM test in adolescent male rats. Each column represents the mean±SEM (8 rats/group). ^****^p < 0.0001 versus Control+Saline group, ^$$$$^p < 0.0001 versus Stress+ Saline group. Correlation analysis across all experimental groups (CS, SS, CD, and SD) revealed a strong negative correlation between total distance travelled and OAT%. **S**: Saline, **D**: Drug (saffron extract), **OAT%**: percentage of time spent in the open arms. **EPM**: Elevated Plus Maze.

The correlation analysis across all experimental groups (CS, SS, CD, and SD) revealed a significant negative association between total distance traveled and OAT% obtained from the EPM test (Pearson’s coefficient = −0.719, P < 0.001) (Fig 9E).

## Discussion

This study investigated ELS in a rat model, exploring the possible therapeutic benefits of saffron extract in mitigating ELS’s adverse metabolic and behavioral effects. ELS paradigms can broadly be categorized into maternal separation models and early aversive or unpredictable stress models. Maternal separation paradigms primarily model early-life deprivation and disruption of maternal care. In contrast, neonatal foot-shock or unpredictable postnatal aversive stress paradigms simulate exposure to distinct, uncontrollable, and physically unpleasant traumatic experiences during a critical developmental stage [6,7]. Importantly, previous studies using early-life unpredictable or foot shock–based stress paradigms have demonstrated long-lasting alterations in emotional and stress-related behaviors, including increased anxiety-like behavior and persistent dysregulation of HPA axis [7–9]. These behavioral phenotypes are accompanied by enduring changes in limbic circuitry, particularly within the amygdala and hippocampus, which are key regions involved in stress regulation [8,9]. Unlike maternal separation paradigms, aversive stress models minimize the confounding influence of variations in maternal caregiving [10,11], providing a more controlled and reproducible approach for examining how early traumatic experiences shape the development of stress-related neural circuits.

Our findings demonstrate that repeated foot‐shock exposure during early life was associated with activation of the HPA axis, as evidenced by elevated basal plasma corticosterone levels in adolescent rats. Previous evidence indicates that ELS induces long-term neuroendocrine reprogramming characterized by increased circulating corticosterone together with reduced glucocorticoid receptor (GR) expression in stress-regulatory brain regions such as the hippocampus, hypothalamic paraventricular nucleus, and frontal cortex [44]. Importantly, Uys et al. demonstrated that early-life trauma increased basal corticosterone while simultaneously decreasing hippocampal GR immunoreactivity, suggesting impaired glucocorticoid-mediated negative feedback regulation of the HPA axis. These findings support the biological plausibility that sustained corticosterone elevation after ELS may be associated with disrupted GR signalling and prolonged stress responsivity [45]. Meanwhile, reduced hippocampal GR expression following impaired maternal care has been linked to epigenetic modifications of the GR promoter, resulting in weakened glucocorticoid-mediated feedback inhibition and prolonged corticosterone secretion [46]. In parallel, maternal separation has been shown to induce a persistent increase in corticotropin-releasing hormone (CRH) and arginine vasopressin (AVP) signalling within the paraventricular nucleus (PVN), further contributing to chronic HPA-axis hyper-responsiveness [47]. In addition, the reviewed literature suggests that ELS may alter glucocorticoid signalling through impaired GR binding/phosphorylation and dysregulation of glucocorticoid-sensitive metabolic pathways. ELS has also been proposed to increase tissue glucocorticoid availability through altered 11β-hydroxysteroid dehydrogenase type 1 (11β-HSD1) activity, thereby amplifying glucocorticoid exposure and contributing to maladaptive HPA axis regulation [44]. Therefore, although GR signalling was not directly measured in the current study, our findings are consistent with previous reports linking ELS-induced corticosterone elevation to impaired glucocorticoid feedback mechanisms. Future studies investigating GR/mineralocorticoid receptor (MR) expression and downstream signalling pathways will be important to further clarify the mechanistic relationship between corticosterone elevation, oxidative stress, and persistent HPA axis dysfunction. Coincident with increased corticosterone, we observed significant increases in MDA and catalase activity alongside depletion of GSH within the amygdala, pancreas, and circulation. It is noteworthy that the increased catalase activity observed in the SS group, despite reduced GSH levels, likely reflects a compensatory enzymatic response to elevated oxidative stress rather than improved antioxidant status [48,49]. Under excessive ROS production, antioxidant enzymes such as catalase are commonly upregulated to maintain redox homeostasis, whereas non-enzymatic antioxidants, including GSH, may become depleted under persistent oxidative challenge [48–50]. Therefore, the concurrent increase in catalase activity and reduction in GSH levels are not contradictory, but rather represent distinct adaptive components of the endogenous antioxidant defence system. Moreover, the reduction in catalase activity following saffron treatment may indicate attenuation of oxidative burden and restoration of redox balance rather than suppression of antioxidant defence [50,51]. Corticosterone excess enhances reactive oxygen species (ROS) production by upregulating nicotinamide adenine dinucleotide phosphate (NADPH) oxidase and impairing mitochondrial electron transport chain function [52,53], leading to lipid peroxidation and protein oxidation in stress‐sensitive tissues. Our results are in line with earlier findings in maternal separation models, which show elevated lipid peroxidation in limbic regions [54,55], and extend these by demonstrating parallel oxidative dysregulation in peripheral metabolic organs. This coordination suggests a systemic redox disturbance that may underlie both central and peripheral consequences of ELS. The long-lasting alterations in brain development, including persistent changes in neural circuit maturation, stress responsivity, and neurobiological signalling systems, may exhibit sex-dependent characteristics, reflecting the modulatory role of sex hormones on neurodevelopmental and stress-related pathways [56–59]. In particular, oestrogens have been shown to exert broad neuroprotective effects, including modulation of synaptic plasticity, cellular resilience mechanisms, and stress-responsive signalling cascades, which may contribute to differential vulnerability or resilience to stress-related outcomes in females [58–60]. Comparative studies in rodent models of ELS have reported sex-dependent differences in stress responsivity, neurobiological adaptations, and behavioral outcomes [57,59,61]. For example, sex-specific alterations in hippocampal and limbic circuit function following early-life stress exposure suggest that males and females may exhibit distinct patterns of neural adaptation depending on brain region and developmental stage [57,61]. Nevertheless, both sexes generally remain susceptible to long-term ELS-induced neurobiological and behavioral alterations, although the magnitude and direction of these effects may vary [56,57]. Accordingly, if females had been included in the present study, the overall pattern of stress-related neurobiological alterations would likely remain evident, but differences in magnitude or regional specificity could be expected due to the modulatory influence of ovarian hormones. In the present study, the exclusive use of male rats reduced variability associated with estrous cycle–dependent hormonal fluctuations and allowed for a more homogeneous experimental baseline, thereby improving internal consistency in this initial investigation.

The elevated corticosterone resulting from ELS may exert direct and indirect impacts on pancreatic β‑cells. Specifically, glucocorticoids bind to GRs within β‑cells, repressing insulin gene transcription and downregulating key transcription factors such as pancreatic and duodenal homeobox 1 (Pdx1) and musculoaponeurotic fibrosarcoma oncogene homologue A (MAFA) that govern insulin synthesis [62]. Concurrently, glucocorticoid-driven oxidative stress—mediated by increased ROS generation via NADPH oxidase activation and mitochondrial dysfunction—overwhelms antioxidant defences within β‑cells, triggering endoplasmic reticulum (ER) stress and unfolded protein responses [63]. The latter could result in the activation of Jun N-terminal kinase (JNK) and C/EBP Homologous Protein (CHOP) pathways, compromising β-cell viability and secretory capacity [64], which may lead to hypoinsulinemia, elevated fasting glucose, and a reduced HOMA‑β index as observed in our experiment. Collectively, these findings suggest a potential link between HPA axis hyperactivity, oxidative imbalance, and impaired glucose regulation. In the central nervous system, the amygdala acts as a critical hub for stress and emotional regulation [65]. The current study demonstrated that ELS elevated oxidative stress markers, reduced insulin content, and simultaneously increased insulin receptor expression in the amygdala, which may indicate the altered insulin signalling/homeostasis. Insulin acting in the central amygdala binds to its receptor, the insulin receptor. It rapidly activates phosphatidylinositol 3-kinase/protein kinase B (PI3K–Akt) signalling, which helps maintain synaptic levels of the α-amino-3-hydroxy-5-methyl-4-isoxazolepropionic acid receptor (AMPAR) subunit GluA1, thereby contributing to emotional regulation [66]. When the insulin receptor together with insulin-like growth factor 1 receptor (IGF-1R) is deleted in the central amygdala (CeA), mice show a 60–70% drop in synaptosomal GluA1 and a marked increase in anxiety-like behaviors, demonstrating that intact amygdala insulin signalling is intrinsically anxiolytic and pro-cognitive [67]. Moreover, Gual et al. demonstrated that increased insulin receptor substrate-1 (IRS-1) Ser307 phosphorylation is closely associated with inhibition of insulin signalling and reduced PI3K activity under stress-related and insulin-resistant conditions [68]. Similarly, Aguirre et al. reported that phosphorylation of IRS-1 at Ser307 blocks insulin receptor interaction and inhibits insulin action [69]. Therefore, assessment of IRS-1 (Ser307) and Akt (Ser473) phosphorylation would indeed provide substantially stronger mechanistic evidence regarding whether chronic ELS induces functional impairment of insulin signalling in the amygdala. Previous studies have also shown that brain insulin signalling exhibits region-specific functional characteristics and may be differentially affected under pathological conditions [70,71]. Because we did not evaluate insulin-related markers in other brain regions, we cannot determine whether the observed alterations are unique to the amygdala or represent a broader neurobiological response to chronic ELS. We propose that future investigations compare insulin signalling pathways across multiple brain regions to determine the spatial specificity of ELS-induced alterations. On the other hand, insulin regulates inhibitory neurotransmission through direct enhancement of gamma-aminobutyric acid (GABA) ergic synaptic strength to exert anxiolytic effects [66,72]. Insulin activates PI3K-dependent pathways and promotes the synaptic localization of postsynaptic GABA-A receptors, thereby enhancing inhibitory neurotransmission within limbic regions such as the amygdala. Accordingly, disruption of insulin signalling may weaken GABAergic inhibitory control, leading to elevated neuronal excitability and the development of anxiety-like behaviors [73]. Therefore, our findings could demonstrate an association between ELS-induced alterations in insulin-related markers and anxiety-like behaviors. However, direct evaluation of GABAergic function was not included in the current study and warrants further investigation in future studies.

It is revealed that heightened ROS and ER stress within amygdala neurons disrupt insulin signalling cascade by activating protein kinase C-θ (PKCθ) and impairing glucose transporter 4 (GLUT4) ‑mediated glucose uptake [15]. The increase in insulin receptor density that we observed may reflect a compensatory mechanism to maintain insulin signalling in response to insufficient insulin levels. Behaviorally, these molecular alterations coincided with anxiety-like phenotypes, including diminished open-arm exploration, increased center zone avoidance, and hyperlocomotion, consistent with dysregulated amygdala circuits. These observations corroborate prior findings that experimentally induced central insulin resistance precipitates anxiety behaviors, highlighting the essential role of intact insulin signalling in emotional homeostasis.

The experimental manipulations, including repeated intraperitoneal saline injections and foot-shock stress exposure, were performed during postnatal days 15–19, whereas behavioral testing was conducted at postnatal day 54, corresponding to late adolescence in rats. Therefore, the observed behavioral alterations likely reflect long-term neurodevelopmental consequences of early-life stress rather than acute procedural effects at the time of experiment. Thus, as previously stated, stress exposure during sensitive developmental periods can induce persistent alterations in HPA axis activity and prefrontal–limbic circuitry, resulting in enduring anxiety-related behavioral changes [74–76]. In addition, late adolescence represents a transitional neurodevelopmental stage during which maturation of prefrontal–limbic networks involved in emotional regulation and stress responsiveness is still ongoing [77]. Consequently, anxiety-like behaviors observed at this age may reflect both early-life neurobiological programming and the heightened neural sensitivity characteristic of this developmental stage [75]. Accordingly, the reduced center exploration seen in the current study, even among the control group (below 10%), may partially result from the combined effects of neonatal stress exposure—caused by repeated IP injections in controls and by repeated IP injections together with foot-shock stress in the stressed group—and heightened neurobehavioral vulnerability during adolescence. Notably, the markedly reduced center exploration in the stress group (<1%), accompanied by increased locomotor activity, is consistent with a robust anxiety-like phenotype characterized by enhanced thigmotaxis and avoidance behavior [78,79]. The similar behavioral pattern observed in both the OFT and EPM further supports this interpretation. In addition, the significant negative correlation between locomotor activity and the anxiety-related parameters observed in our study reinforces the presence of an anxiety-like phenotype, indicating that increased activity occurred in parallel with enhanced avoidance-related behavior. Moreover, early-life stress during sensitive developmental windows may disrupt maturation of prefrontal–limbic circuitry, leading to persistent hypervigilance and avoidance-related behaviors manifested as increased thigmotaxis and reduced center/open-arm exploration in the OFT and EPM [80,81]. Importantly, all experimental groups were evaluated under identical conditions and at the same developmental stage, supporting the validity of between-group comparisons.

Administration of saffron extract prior to stress exposure may contribute to the normalization of corticosterone levels, redox biomarkers, metabolic parameters, and anxiety indices compared to control levels. Preclinical studies have demonstrated that Crocus sativus constituents—crocin and safranal—can attenuate HPA axis dysregulation by modulating CRH release and enhancing negative feedback at GRs [82–84]. Previous experimental and clinical studies have demonstrated that these compounds exert anxiolytic, antidepressant, antioxidant, and neuroprotective properties through multiple complementary mechanisms [51,85,86]. One important mechanism involves modulation of neurotransmitter systems associated with emotional regulation. Crocin and safranal have been reported to influence serotonergic, dopaminergic, glutamatergic, and GABAergic neurotransmission [85–87]. In particular, safranal has shown anxiolytic-like effects that may partly involve interaction with the GABA -A-benzodiazepine receptor complex, while crocin has been associated with modulation of serotonin and dopamine signalling pathways [3,4]. These effects may contribute to the behavioral improvements observed in stress-related conditions. The potent free radical‑scavenging activities of crocin and safranal restore GSH pools and reduce lipid peroxidation, thereby alleviating oxidative and ER stress in both central and peripheral tissues [83,88]. Moreover, crocin enhances endogenous enzymatic antioxidant defenses such as superoxide dismutase (SOD) and catalase activity [51,89]. Because oxidative stress is strongly implicated in both insulin signalling impairment and anxiety-related neuropathology, the antioxidant properties of saffron may represent an important mechanism underlying its protective effects in the present study. Furthermore, several studies suggest that crocin may exert neuroprotective actions through regulation of intracellular signaling pathways involved in neuronal survival and synaptic plasticity, including the PI3K/Akt, brain-derived neurotrophic factor (BDNF)/ cAMP-response element binding protein (CREB), and mitogen-activated protein kinases (MAPK) pathways [5,6]. These pathways are closely linked to stress adaptation, neuronal resilience, and insulin signalling regulation. In addition, saffron has been reported to augment insulin signalling and glucose uptake by enhancing GLUT4 translocation and ameliorating oxidative damage in β‑cells [90]. In this study, the administration of saffron extract before stress exposure led to the reversal of ELS-induced phenotypes, underscoring saffron’s capacity to target multiple intersecting pathways—HPA control, redox balance, ER stress, and insulin sensitivity—offering a multifaceted therapeutic approach. The present study did not directly investigate the individual contributions of crocin or safranal, nor did it evaluate specific neurotransmitter or intracellular signalling pathways. Therefore, mechanistic conclusions remain limited.

It should be noted that the control pups were also subjected to repeated handling and temporary separation from the dam during the pre-weaning period as part of the habituation procedure to the communication box. Although these procedures were necessary to minimize novelty-induced stress and to standardize experimental conditions across groups, repeated maternal separation and handling may themselves constitute a mild early-life stressor. However, the duration of separation used in the present study was substantially shorter than established maternal separation stress paradigms reported in the literature. Previous studies in rats have demonstrated that a minimum separation duration of approximately 3 hours is generally required to induce robust long-term stress responses, including elevated ACTH and corticosterone levels in adulthood [91]. Therefore, while a mild stress effect associated with handling and temporary separation cannot be completely excluded, the separation protocol used in the current study was considerably less intensive than classical maternal separation paradigms. Nevertheless, the significant differences observed between control and stressed animals suggest that foot-shock exposure produced effects beyond those associated with handling and temporary separation alone.

### Limitations

This study has several limitations. First, the experiments were conducted exclusively in male Wistar rats using a foot-shock model of ELS, which may limit the generalizability of the findings to females or to other stress paradigms. Second, all outcomes were assessed during adolescence, and extrapolation to other developmental stages or to humans should be interpreted with caution, due to interspecies differences. In addition, mechanistic insights remain limited, as key pathways—including GR/MR signalling, IRS-1 and Akt phosphorylation, GABAergic function, and the specific contributions of crocin and safranal—were not directly evaluated. Furthermore, the absence of comparisons between adolescent and adult offspring restricts conclusions regarding developmental specificity. Another potential limitation is that control animals were also exposed to repeated handling and brief maternal separation during habituation procedures, which may represent a mild form of ELS. Although this approach was used to standardize experimental conditions, its potential influence cannot be entirely excluded. Future studies should address these limitations by incorporating both sexes, multiple developmental time points, detailed mechanistic analyses, and improved experimental designs.

## Conclusion

In conclusion, ELS was associated with increased HPA axis activity, systemic and amygdala oxidative imbalance, impaired glucose homeostasis, and anxiety-like behaviors in adolescent male rats. These alterations were accompanied by reduced insulin content and altered insulin receptor protein levels in the amygdala, suggesting a possible disruption of central insulin signalling pathways. Importantly, pretreatment with saffron extract was associated with attenuation of these alterations, accompanied by normalization of corticosterone levels, improvement of oxidative balance, and amelioration of metabolic and behavioral parameters. Overall, the findings highlight a close association between oxidative stress, HPA axis dysregulation, metabolic dysfunction, and emotional disturbances following ELS and suggest the potential of saffron extract as a multi-target intervention. Future studies are required to establish the underlying mechanisms and causal relationships.

## Supporting information

S1 File Final excel. (XLSX)
